# Nuclear and mitochondrial genomes of *Polylopha cassiicola*: the first assembly in Chlidanotinae (Tortricidae)

**DOI:** 10.1038/s41597-024-03255-7

**Published:** 2024-04-23

**Authors:** Fangyuan Yang, Li-Jun Cao, Jin-Cui Chen, Wei Song, Yuzhu Yu, Shu-Jun Wei

**Affiliations:** 1 Institute of Plant Protection, Beijing Academy of Agriculture and Forestry Sciences, Beijing, China; 2 Guangxi National Qinlian Forest Farm, Guangxi, China

**Keywords:** Genome, Sequence annotation

## Abstract

Tortricidae is one of the largest families in Lepidoptera, including subfamilies of Tortricinae, Olethreutinae, and Chlidanotinae. Here, we assembled the gap-free genome for the subfamily Chlidanotinae using Illumina, Nanopore, and Hi-C sequencing from *Polylopha cassiicola*, a pest of camphor trees in southern China. The nuclear genome is 302.03 Mb in size, with 36.82% of repeats and 98.4% of BUCSO completeness. The karyotype is 2n = 44 for males. We identified 15412 protein-coding genes, 1052 tRNAs, and 67 rRNAs. We also determined the mitochondrial genome of this species and annotated 13 protein-coding genes, 22 tRNAs, and one rRNA. These high-quality genomes provide valuable information for studying phylogeny, karyotypic evolution, and adaptive evolution of tortricid moths.

## Background & Summary

Tortricidae, the leafroller moths, is one of the largest families of Lepidoptera (butterflies and moths)^1^, including numerous notorious economic pests such as the spruce budworm, *Choristoneura fumiferana*^2^, oriental fruit moth *Grapholita molesta*^3^ and codling moth, *Cydia pomonella*^4^. The two main subfamilies are Tortricinae and Olethreutinae, which are relatively young^5^, comprising over 95% of tortricid species. Genomes of many species in these two subfamilies have been determined^6^, revealing an ancestral sex chromosome-autosome fusion and two subsequent autosome fusions relative to the ancestral karyotype of Lepidoptera^7^. Compared to the two successful subfamilies, the relict subfamily Chlidanotinae is much more limited in distribution range, host range, species richness, and population size. Species of this subfamily are mainly distributed in tropical regions, indicating varied climatic adaptability compared to species of the other subfamilies. Thus, this group can provide valuable insights into the phylogeny and pest adaptation and evolution of Tortricidae. However, no genome has been assembled for species of Chlidanotinae.

Here, we present the first chromosome-level genome assembly and annotation in the Olethreutinae using high-coverage long-read and Hi-C sequencing from *Polylopha cassiicola*^8^. This species is mainly distributed in the southern coastal regions of China and Southeast Asia. It is a pest of trees *Cinnamomum cassia* and *C. camphora*. We also assembled the mitochondrial genome of this species from the Illumina short sequencing reads. These genomes are expected to provide information for understanding the phylogeny, karyotypic, and adaptive evolution of Tortricidae.

## Methods

### Sample collection and sequencing

*P. cassiicola* larvae were collected from the tops of *C. camphora* in Guangxi, China. The larvae were reared in the laboratory to pupae and adults for genomic and transcriptome sequencing. Three individuals were used for three types of genome sequencing: one male pupae for Nanopore long-read sequencing, one male pupae for Illumina short-read sequencing, and one female adult for Hi-C sequencing. In addition, four larvae were used for RNA sequencing. Nucleic acid extraction and sequencing libraries was contracted by BerryGenomic (Beijing, China). Methods for nucleic acid extraction, platforms for sequencing, and sequencing outputs are provided in Table 1.

**Table 1:** Methods and outputs for sequencing experiments.

Experiment	Method/Platform	Manufacturer	Insertion size	Output	Coverage
DNA extraction	Magnetic bead method	Invitrogen, Thermo Fisher Scientific, USA	NA	NA	NA
RNA extraction	TRIzol reagent	Thermo Fisher Scientific, USA	NA	NA	NA
Short-read seq	NovaSeq 6000; paired-end	Illumina, USA	350 bp	68.7 Gb	115×
Long-read seq	PromethION	Oxford Nanopore Technologies, UK	N50 = 16.7 Kb	117.6 Gb	196×
Hi-C seq	NovaSeq 6000; paired-end; digested by *MboI*	Illumina, USA	350 bp	178.1 Gb	297×
RNA seq	NovaSeq 6000; paired-end	Illumina, USA	350 bp	16.3 Gb	NA

NA, not available.

**Table 2:** Statistics of repeat elements and non-coding RNAs in *Polylopha cassiicola* genome.

Item	Number	Length (bp)	Content (%)
SINEs	5219	354577	0.12
LINEs	143490	16636355	5.51
LTR elements	35392	11777710	3.9
DNA transposons	23636	4498331	1.49
Rolling-circles	315217	39695809	13.14
Unclassified repeats	235177	34162427	11.31
Satellites	17	2222	0
Simple repeats	72367	3737369	1.24
Low complexity repeats	7520	355233	0.12
rRNAs	67	46500	0.015
tRNAs	1052	78802	0.026

SINEs, short interspersed nuclear elements; LINEs, long interspersed nuclear elements; LTR, long terminal repeat.

### Genome assembly

**Fig. 1:**
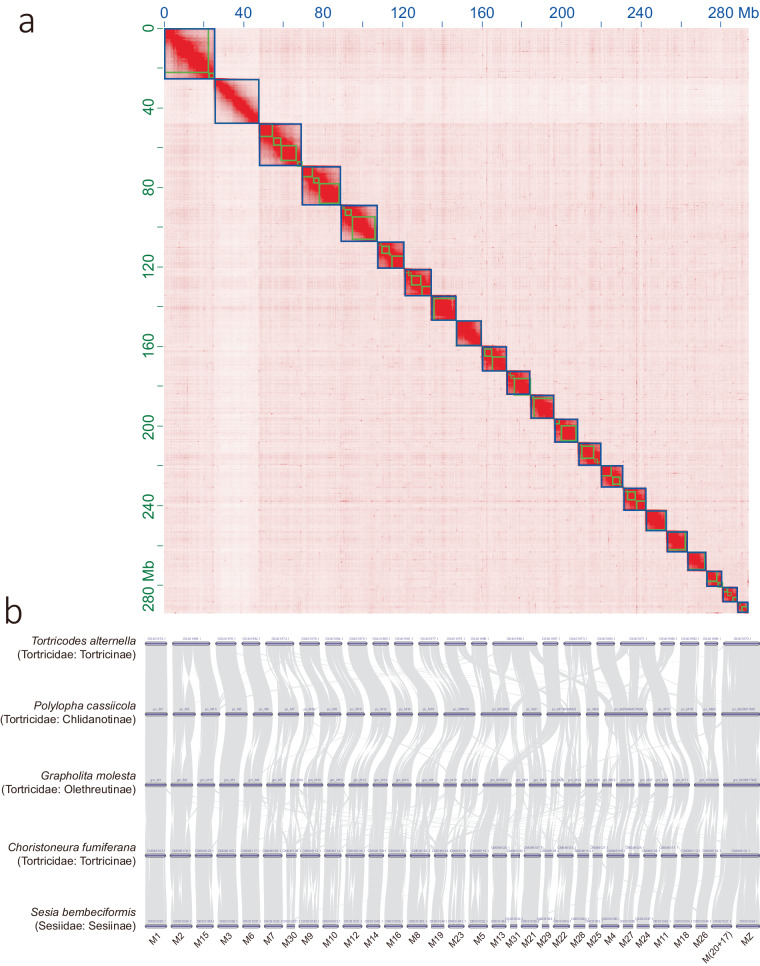
Genomic feature of nuclear genome of *Polylopha cassiicola*. (**a**) Hi-C contact matrix of 22 putative chromosomes. (**b**) Synteny among four tortricid species from four subfamilies and an outgroup. The labels at the bottom marked the ancestral linkage groups of Lepidoptera^6^.

The Nanopore long reads were assembled into 76 contigs using NextDenovo 2.5.2 (https://github.com/Nextomics/NextDenovo) with parameters: “read_cutoff = 4k, genome_ size = 400 m, nextgraph_options = -a 1”. Redundant sequences in contigs were removed using Purge_dups^9^. The cleaned contigs containing 65 sequences were then assembled to chromosome-level using Hi-C information. In this analysis, we mapped the Hi-C reads to cleaned contigs using BWA^10^ with options: “mem -SP5”, anchored contigs using YaHS 1.2a.1^11^ with option: “-e GATC”, and manually adjusted using Juicerbox 1.22.01^12^. We removed the contigs that did not have any contact information with the chromosomes, which could be from potential contamination, such as symbiotic microbes. At last, the chromosomal-level genomic sequences were subjected to two rounds of long-read polishing and two rounds of short-read polishing using Nextpolish 1.4.1^13^. The obtained *P. cassiicola* genome is 302.03 Mb in size and contains 21 autosomes and one Z sex-chromosome (Fig. 1a).

**Fig. 2:**
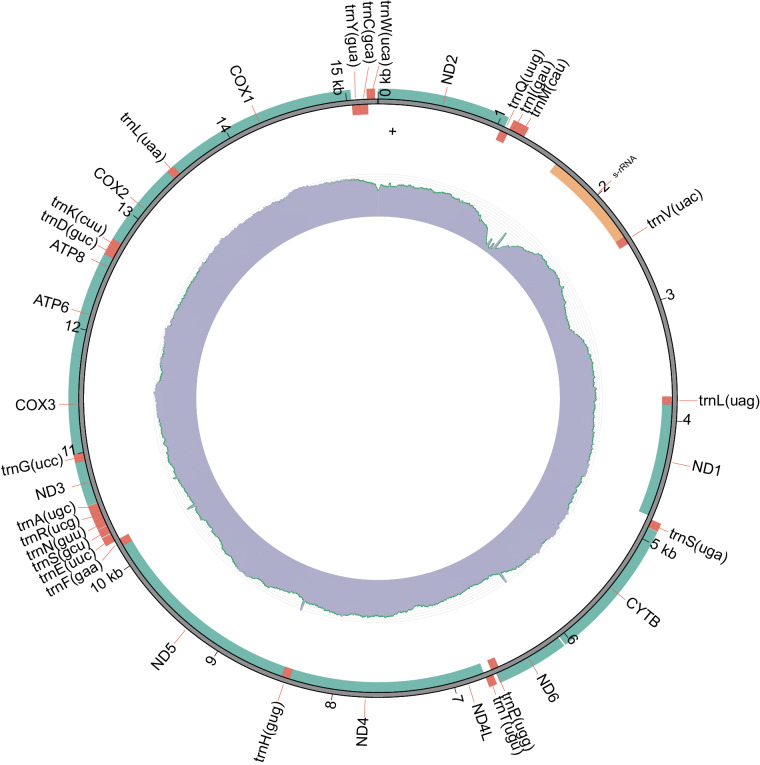
Distribution of annotated genes on mitochondrial genome. The inner ring shows the relative read coverage.

We also assembled mitochondrial genome using MitoZ 3.6^14^ based on the short-reads. In the mitochondrial genome, we identified 13 protein-coding genes, 22 tRNAs, and 1 rRNA (Fig. 2).

### Genome synteny

We analysed the chromosomal synteny between *P. cassiicola* and three other species from Tortricidae and one from Sesiidae: *Choristoneura fumiferana* (Tortricidae: Tortricinae)^2^, *Grapholita molesta* (Tortricidae: Olethreutinae)^3^, *Tortricodes alternella* (Tortricidae: Tortricinae; NCBI GenBank assembly: GCA_947859335.1^15^), and *Sesia bembeciformis* (Sesiidae: Sesiinae)^16^. Synteny analysis was conducted using MSCANX pipeline in JCVI utility libraries^17^. We assigned names of the ancestral linkage group in Lepidoptera^6^ (Merian elements, M1-31 and MZ) based on chromosomal homology. The results show different patterns of chromosomal fusion in species *T. alternella* and *P. cassiicola* (Fig. 1b).

### Repeat element and non-coding RNA annotation

Repeat elements were detected using RepeatMasker 4.1.5^18^ with options “-no_is -norna -xsmall -q”. This analysis was conducted against three databases: Repbase (http://www.girinst.org), Dfam database1 specific to Arthropoda, and a species-specific repeat library constructed using RepeatModeler2^19^. Transfer RNA (tRNA) was predicted by tRNAscanSE 2.0.12^20^ with default parameters, and ribosome RNA (rRNA) was predicted using Barrnap 0.9 (https://github.com/tseemann/barrnap). In the *P. cassiicola* genome, 36.82% of bases were annotated as repeat elements (Table 2). We identified 67 rRNAs, and 1052 tRNAs (Table 2).

**Table 3:** Statistics of *Polylopha cassiicola* assemblies.

Item	Contig	Purged contig	Hi-C raised scaffold	Polished scaffold
No. of contigs	76	65	22	22
Size (Mb)	297.20	294.45	294.46	302.03
N50 (Mb)	8.54	8.54	12.96	13.19
GC content	35.16%	35.12%	35.12%	35.14%
Single-copy BUSCOs	94.7%	94.8%	94.9%	98.1%
Duplicated BUSCOs	0.5%	0.3%	0.3%	0.3%
Fragmented BUSCOs	2.2%	2.2%	2.2%	0.3%
Missing BUSCOs	2.6%	2.7%	2.6%	1.3%

### Gene prediction and functional annotation

Gene structure was predicted using an ab initio method, Helixer^21^, with options: “–subsequence-length 320760–batch-size 6”, and with a pre-trained model for invertebrate “invertebrate_v0.3_m_0200”. Gene function, Gene Ontology (GO), and Kyoto Encyclopedia of Genes and Genomes (KEGG) items for predicted genes were annotated using eggNOG-Mapper^22^ web tools, against the eggNOG Database 5. A total of 15412 protein-coding genes were predicted, in which 12671 genes were functionally annotated.

## Data Records

The Nanopore reads, Illumina reads, Hi-C reads, and RNA reads for *P. cassiicola* genome assembly were deposited at NCBI under Sequence Read Archive under accession number SRP479759^23^. The nuclear and mitochondrial genome assemblies were deposited in Genbank under accession number GCA_038024825.1^24^. The genome annotation files are available in Figshare^25^ at 10.6084/m9.figshare.24902046.

## Technical Validation

To validate the accuracy of the final genome assembly, we mapped the Illumina short reads and Nanopore long reads to the *P. cassiicola* genome using Minimap2^26^ with option “-ax sr” for short reads and option “-ax map-ont” for long reads. The mapping rates for the short reads and long reads were calculated using Samtools^27^. Analysis revealed 96.38% and 98.73% mapping rates for the short and long reads, respectively. We examined the coverage of short reads along the mitochondrial genome and showed 100% coverage (Fig. 1b).

Completeness of the assembly and gene prediction were evaluated using BUSCO 5.4.7^28^ with lepidoptera_odb10 database. In this analysis, BUSCO examined the states and proportions of 5,286 single-copy orthologous of Lepidoptera in our genome assembly: single-copy (S), duplication (D), fragment (F), and missing (M). The analyses showed completeness ranging 95.1%–98.4% for each assembly stage (Table 3), and 97.8% for predicted gene set: “C: 97.8% [S: 97.2%, D: 0.6%], F: 0.9%, M: 1.3%”. Quality of gene prediction was manually evaluated using RNA-seq data. Specifically, RNA-seq reads were mapped to the genome using Hisat^29^ and Samtools^27^. We imported the obtained BAM file and annotation file into the IGV browser^30^. Based on manual examination, we found that the machine learning-based annotation method has predicted a near-complete gene structure. These results indicate that we have obtained a high-quality assembly and annotation for *P. cassiicola* genome.

## Data Availability

No custom scripts or code were used in this study.

## References

[1] van der Geest, L. P. S. & Evenhuis, H. H. *Tortricid Pests: Their Biology, Natural Enemies, and Control*. vol. 5 (Elsevier, 1991).

[2] Béliveau C (2022). The spruce budworm genome: reconstructing the evolutionary history of antifreeze proteins. Genome Biol. Evol..

[3] Cao L-J (2022). Population genomic signatures of the oriental fruit moth related to the Pleistocene climates. Communciations Biol..

[4] Wan F (2019). A chromosome-level genome assembly of *Cydia pomonella* provides insights into chemical ecology and insecticide resistance. Nat. Commun..

[5] Fagua G, Condamine FL, Horak M, Zwick A, Sperling FAH (2017). Diversification shifts in leafroller moths linked to continental colonization and the rise of angiosperms. Cladistics.

[6] Wright, C. J., Stevens, L., Mackintosh, A., Lawniczak, M. & Blaxter, M. Comparative genomics reveals the dynamics of chromosome evolution in Lepidoptera. *Nat. Ecol. Evol*. 1–14 10.1038/s41559-024-02329-4 (2024). 10.1038/s41559-024-02329-4 PMC11009112 38383850

[7] Šíchová J, Nguyen P, Dalíková M, Marec F (2013). Chromosomal evolution in tortricid moths: conserved karyotypes with diverged features. PLoS ONE.

[8] Nasu Y (2006). *Lopharcha moriutii*, sp. nov. and *Polylopha cassiicola* Liu & Kawabe (Lepidoptera, Tortricidae, Chlidanotinae, Polyorthini) from Thailand and Hong Kong. Zootaxa.

[9] Guan D (2020). Identifying and removing haplotypic duplication in primary genome assemblies. Bioinformatics.

[10] Li H, Durbin R (2009). Fast and accurate short read alignment with Burrows–Wheeler transform. Bioinformatics.

[11] Zhou C, McCarthy SA, Durbin R (2023). YaHS: yet another Hi-C scaffolding tool. Bioinformatics.

[12] Durand NC (2016). Juicer provides a one-click system for analyzing loop-resolution Hi-C experiments. Cell Syst..

[13] Hu J, Fan J, Sun Z, Liu S (2020). NextPolish: a fast and efficient genome polishing tool for long-read assembly. Bioinformatics.

[14] Meng G, Li Y, Yang C, Liu S (2019). MitoZ: a toolkit for animal mitochondrial genome assembly, annotation and visualization. Nucleic Acids Res..

[15] Wellcome Sanger Institute (2023). Genbank.

[16] Boyes, D. & Langdon, W. B. V. The genome sequence of the Lunar Hornet, *Sesia bembeciformis* (Hübner 1806). *Wellcome Open Res* **8**, (2023). 10.12688/wellcomeopenres.19111.1 PMC10568212 37840882

[17] Tang H (2008). Synteny and Collinearity in Plant Genomes. Science.

[18] Tarailo-Graovac M, Chen N (2009). Using RepeatMasker to identify repetitive elements in genomic sequences. Curr. Protoc. Bioinforma..

[19] Flynn JM (2020). RepeatModeler2 for automated genomic discovery of transposable element families. Proc. Natl. Acad. Sci..

[20] Chan PP, Lin BY, Mak AJ, Lowe TM (2021). tRNAscan-SE 2.0: improved detection and functional classification of transfer RNA genes. Nucleic Acids Res..

[21] Stiehler F (2021). Helixer: cross-species gene annotation of large eukaryotic genomes using deep learning. Bioinformatics.

[22] Cantalapiedra CP, Hernández-Plaza A, Letunic I, Bork P, Huerta-Cepas J (2021). eggNOG-mapper v2: Functional annotation, orthology assignments, and domain prediction at the metagenomic scale. Mol. Biol. Evol..

[23] (2024). NCBI Sequence Read Archive.

[24] (2024). Genbank.

[25] Yang F, Wei S-J (2023). figshare.

[26] Li H (2018). Minimap2: pairwise alignment for nucleotide sequences. Bioinformatics.

[27] Danecek P (2021). Twelve years of SAMtools and BCFtools. GigaScience.

[28] Manni M, Berkeley MR, Seppey M, Simão FA, Zdobnov EM (2021). BUSCO update: Novel and streamlined workflows along with broader and deeper phylogenetic coverage for scoring of eukaryotic, prokaryotic, and viral genomes. Mol. Biol. Evol..

[29] Kim D, Paggi JM, Park C, Bennett C, Salzberg SL (2019). Graph-based genome alignment and genotyping with HISAT2 and HISAT-genotype. Nat. Biotechnol..

[30] Robinson JT (2011). Integrative genomics viewer. Nat. Biotechnol..

